# Combined transcriptomic and lipidomic analysis reveals aberrant lipid metabolism in central nervous system hemangioblastomas

**DOI:** 10.1038/s41598-020-80263-8

**Published:** 2021-01-14

**Authors:** Qiguang Wang, Wenke Liu, Si Zhang, Zuoyu Liang, Linhong Jiang, Aiqin Xue, Xiaobo Cen, Qian Bu

**Affiliations:** 1 West China School of Public Health and West China Fourth Hospital, Sichuan University, Chengdu, 610041 China; 2 Department of Neurosurgery, West China Hospital, Sichuan University, Chengdu, 610065 China; 3 National Chengdu Center for Safety Evaluation of Drugs, State Key Lab of Biotherapy/Collaborative Innovation Center of Biotherapy, West China Hospital, West China Medical School, Sichuan University, Chengdu, 610041 China; 4 Department of Pathology, West China Hospital, Sichuan University, Chengdu, 610065 China

**Keywords:** Biochemistry, Cancer

## Abstract

Peritumoral cysts are commonly detected in the central nervous system tumors, especially hemangioblastomas (HBs). However, the molecular mechanisms driving their formation and propagation are still unknown. We conducted an integrated lipidomics and transcriptomics analysis on solid and cystic HB samples in order to elucidate the changes in the lipid profile and expression of lipid metabolism-related genes during cyst formation. Transcriptomic analysis revealed differential expression of several genes between the solid and cystic HBs, and those associated with lipid metabolism, such as ADCY4, MGLL, ACOT2, DGKG, SHC1 and LPAR2, were markedly dysregulated in the cystic HBs. The lipidomic analysis further showed a significant reduction in the abundance of triacylglycerol, ceramide, lysophosphatidylcholine and lysophosphatidylethanolamine, and an increase in phosphatidylcholine and phosphatidylethanolamine levels in the cystic HBs. Furthermore, bioinformatics analysis revealed altered lipid biosynthesis, glycerophospholipid metabolism and phospholipase activity in the cystic HBs. Taken together, our findings indicate that cyst formation in HBs is related with aberrant lipid metabolism.

## Introduction

The clinical symptoms of several central nervous system (CNS) tumors can be attributed to the formation of peritumoral cysts in the brain and spinal cord^1^. However, the molecular mechanisms underlying their formation and propagation are still unknown. Hemangioblastomas (HBs) are highly vascularized tumors that comprise up to 3% of all brain tumors, and mainly occur in the cerebellum (~ 60%)^2^. HBs are defined as grade I according to the 2016 World Health Organization Classification of Tumors of CNS^3^, and predominantly consist of pericytes, endothelial cells and stromal cells^4^. Radiologically, they are frequently associated with peritumoral cysts^5,6^. Despite their benign nature, HBs are associated with significant morbidity and mortality due to mass effect of the primary tumor or the peri-tumoral cysts^7^. Studies show that more than 70% of HBs-related neurological impairment is caused by peri-tumoral cysts^8,9^. These characteristics make HBs a promising model for studying the formation of peritumoral and intra-tumoral cysts.

Studies show that peri-tumoral cysts form due to the leakage of plasma-ultrafiltrate through the permeable tumor vessels, and its subsequent accumulation in the surrounding CNS tissues^10,11^. Vascular endothelial growth factor (VEGF), a key regulator of neoplastic vascularization in HBs^12,13^, increases vascular permeability and facilitates cyst formation in HBs. In contrast, some studies have reported similar VEGF expression levels in the solid and cystic HBs, indicating involvement of other factors as well. For example, aquaporin (AQP-1) and Ang/Tie signaling have also been implicated in HB cyst formation^10,14^.

Lipids are the primary macromolecules constituting the structural components of CNS. They regulate stability/fluidity of cellular membranes, neuronal energy production, vesicular transport and signaling, and dysregulated lipid metabolism in various tissues is associated with cancer progression^15–17^. Since bioactive lipids and lipid-modified proteins can drive tumor pathogenesis via multiple signaling networks, lipidomics, or the characterization of lipids within a given cell or organism, has recently gained attention in cancer research^18^. For instance, lipidomics analysis of human breast tissues revealed that changes in phospholipids are associated with breast cancer progression and patient survival^19^. Furthermore, high-throughput platforms for the systematic analysis of multi-dimensional omics data have revealed previously unknown connections between molecular signatures, including lipid-gene networks^20^.

We surmised that delineation of the altered lipid metabolism in HBs may provide new insights into their initiation and progression, neo-angiogenesis and peri-tumoral cyst formation. To this end, we analyzed integrated lipidomic and transcriptomic data of CNS HBs samples, and detected significant metabolic changes between solid and cyst-associated HBs, along with the associated pathways.

## Results

### Clinical features of HBs patients

**Figure 1:**
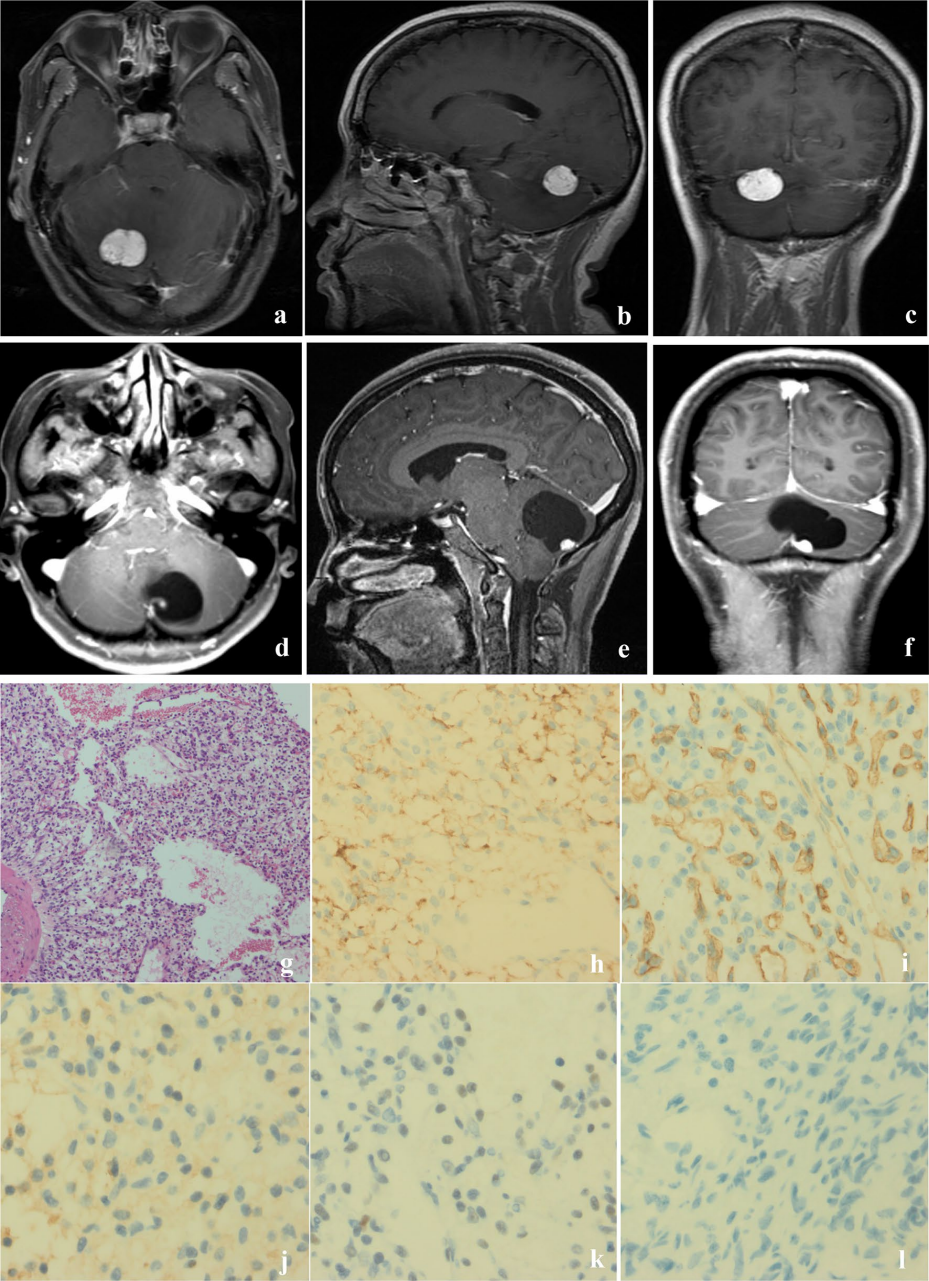
Radiographic and immunohistochemical features of HBs. (**a**–**c**) Axial, sagittal and coronal contrast enhanced T1-weighted MRI of a solid HB showing well-circumscribed solid mass in the right cerebellar hemisphere. (**d**–**f**) Axial, sagittal and coronal contrast enhanced T1-weighted MRI of a cystic HB showing a non-enhanced cystic mass in the right cerebellar hemisphere with an enhancing mural nodule. The enhanced tumor nodule contained tumor cells, and surrounding cyst consisted of plasma ultrafiltrates. (**g**) HE staining demonstrated vascular and stromal cells in cystic HBs, ×100. Immunohistochemical staining of cystic HBs revealed tumor cells staining positive for carbonic anhydrase IX (CAIX) (**h**); endothelial cell marker (CD34) (**i**); 2-phospho-D-glycerate hydrolase (NSE) (**j**); SOX9 (**k**) and negative for phosphoenolpyruvate Carboxykinase (PCK) (**l**), ×400.

Eleven patients including 6 males and 5 females with mean age of 47.9 years (31–70 years) were recruited in the study. The mean duration of symptoms, including dizziness, headache and vomiting, was 5.15 months (range 0.3–12 months). Two patients had genetic and/or clinical evidence of VHL disease, while the others had a sporadic disease. The general features of the patients are summarized in Table 1. Based on the Gd-enhanced MR images and intraoperative findings, 8 HBs showed a macroscopic cystic pattern (Fig. 1a–c), while solid pattern was observed in 3 HBs (Fig. 1d–f). HE and immunohistochemical staining showed extensive vascularization and presence of stromal cells in the cystic HBs (Fig. 1g–l). Numerous CD31 + thin-walled microvessels were seen, indicating reactive angiogenesis (Fig. 1h). All tumors were positive for neuron-specific enolase (NSE) and negative for GFAP (Table S2). In addition, Ki67 staining demonstrated mitosis in some cells, and an overall low proliferation rates of 1–30% (Table S2). Immunohistochemical staining results are summarized in Table S2.

**Table 1:** Summary of clinical characteristics of HBs patients in our study.

Case no	Age (years)	Gender	Duration (months)	Symptoms & signs	Tumor characteristics	Location	Size (Dmax)	VHL disease
1	70	Male	4	Dizziness	Cystic	CH	5	N
2	61	Female	0.3	Headache, dizziness	Solid	CH	2.6	N
3	40	Female	12	Dizziness	Cystic	CH	3.5	N
4	45	Male	0.75	Dizziness	Cystic	CPA	5	Y
5	53	Female	1.5	Headache	Solid	CH	2.5	N
6	31	Female	8	Headache, dizziness	Cystic	CH	5.4	N
7	41	Female	12	Headache, dizziness	Cystic	CH	4.1	N
8	43	Male	6	Dizziness	Solid	CH	1.8	N
9	54	Male	No	No symptoms	Cystic	CH	3	N
10	48	Male	6	Headache, vomiting	Cystic	CH&CV	4.6	N
11	41	Male	12	Headache, dizziness	Cystic	CH&CV	5	Y

*CH* cerebellar hemisphere, *CV* cerebellar vermis, *CPA* cerebellopontine angle, *VHL* Von Hippel–Lindau syndrome, *Dmax* maximum diameter (cm), *Y* yes, *N* No.

### Solid and cystic HBs show different gene expression profiles

**Figure 2:**
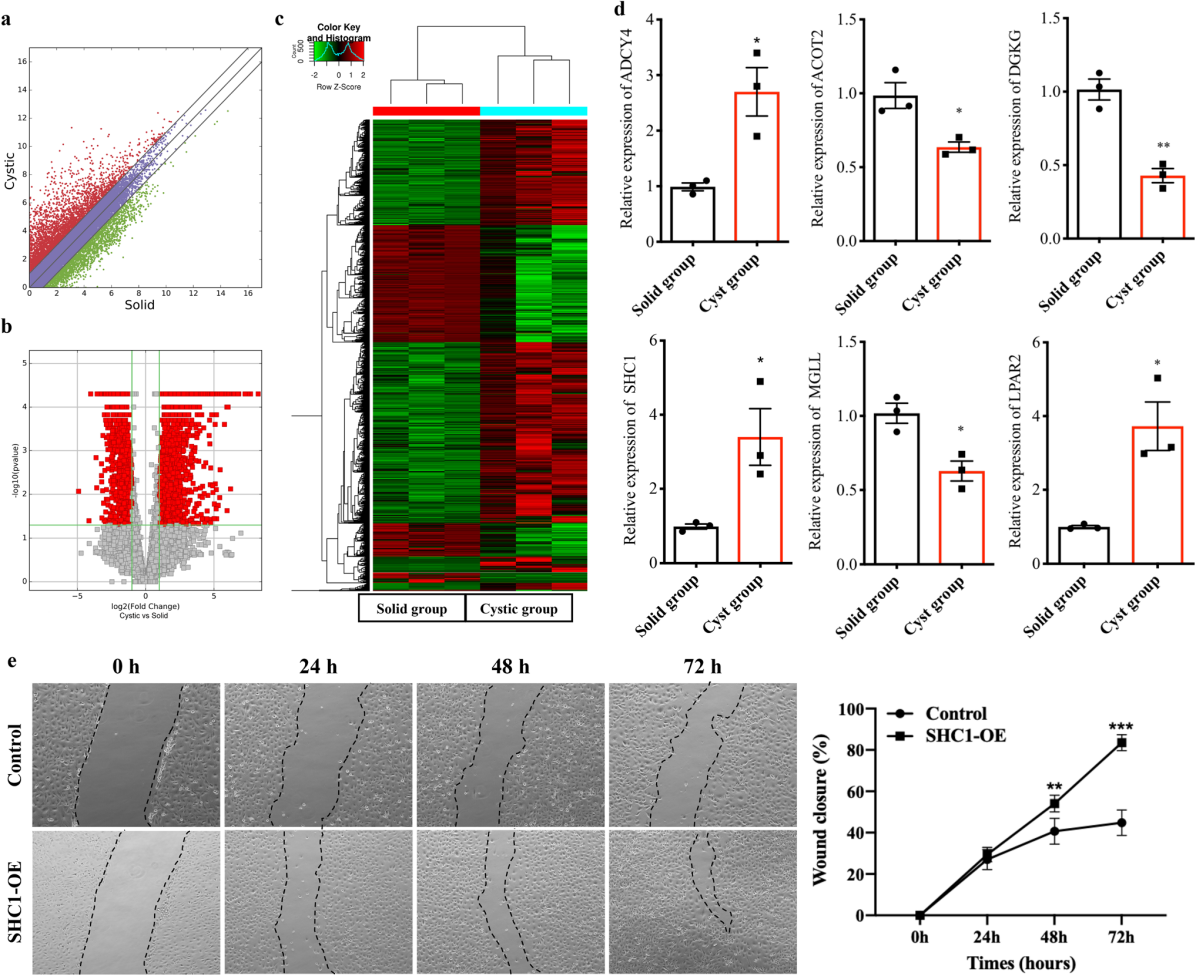
Differential gene expression profiles in solid and cystic HBs. (**a**) Scatter plots showing the mRNA expression profiles of solid and cystic HBs. (**b**) Volcano plots showing the mRNA expression profiles in solid and cystic HBs. Red points indicate the different expression of mRNAs (fold change > 1.5, *p* < 0.05). (**c**) Heat map of DEGs between the two groups. (**d**) RT-qPCR results validating the mRNA levels of 6 selected genes. *p < 0.05, **p < 0.01. (**e**) The migration ability of HUVECs in control and SHC1-OE cells was tested by the scratch wound assay. Representative images were shown in the left panels. Quantitative analysis of the wound closure was shown in the right panels. n = 6, **p < 0.01, ***p < 0.001.

To determine the lipidomic alterations between solid and cystic HBs, we assessed the expression of genes involved in lipid metabolism (Fig. 2a), and identified 3334 DEGs between the two groups on the basis of fold change ≥ or ≤ 2 and *p* = 0.05 (Fig. 2b), of which 2190 were up-regulated and 1144 were down-regulated in the cystic HBs (Supplementary Table S3). Furthermore, supervised hierarchical cluster analysis showed that the DEGs were able to distinguish between the cystic and solid tumors (Fig. 2c).

### The DEGs between solid and cystic HBs are related to lipid metabolism

**Figure 3:**
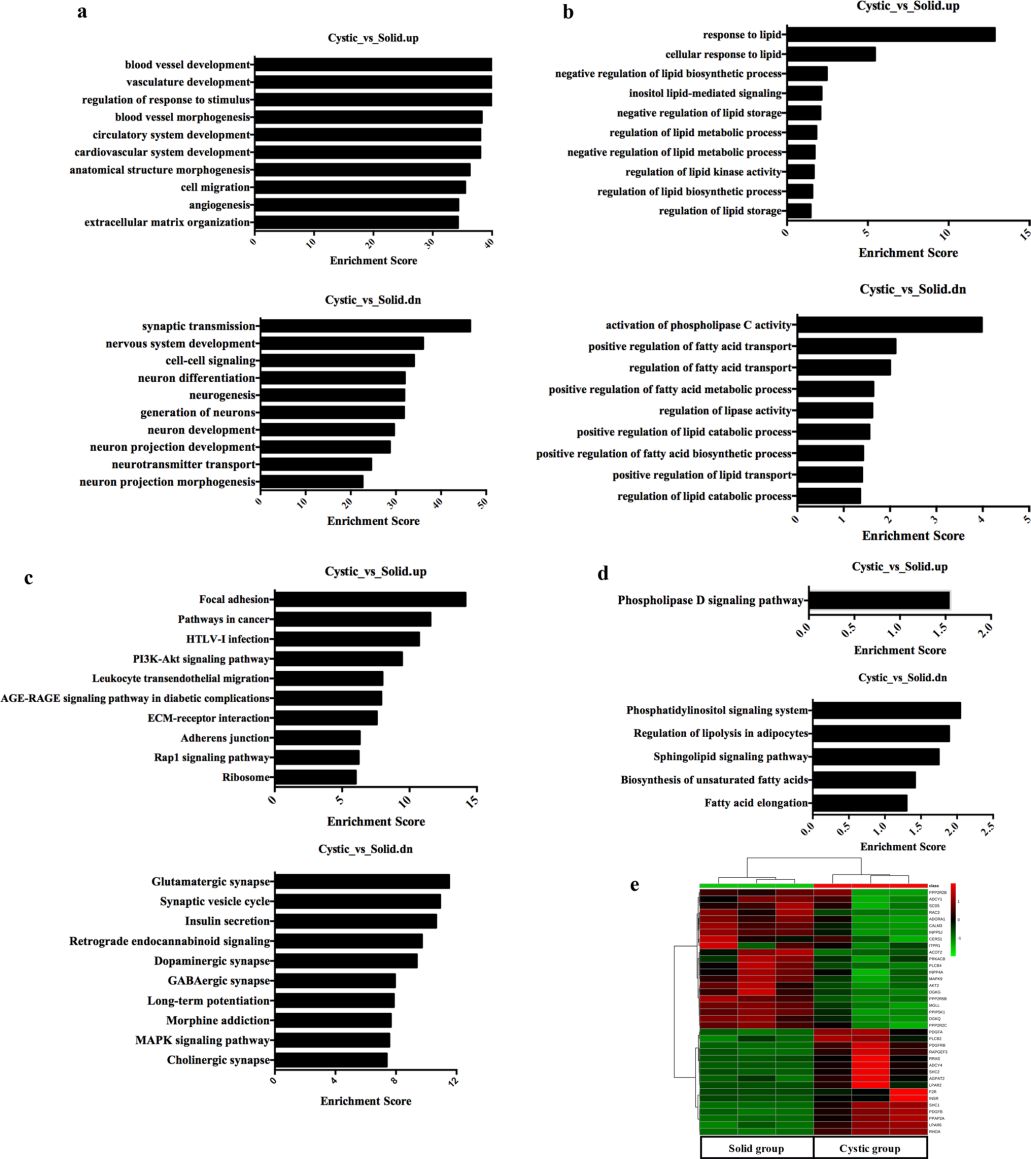
GO and KEGG analysis of DEGs. (**a**) Top 10 significantly enriched biological processes (BP) among DEGs in solid and cystic HBs. (**b**) GO analysis of lipid metabolism-related DEGs. (**c**) Top 10 significantly enriched KEGG pathways among DEGs in solid and cystic HBs. (**d**) KEGG pathway analysis of the lipid metabolism-related DEGs. (**e**) Heat map of DEGs according to lipid metabolism in solid and cystic HBs.

The DEGs were functionally annotated using GO analysis (Supplementary Table S4). The significantly enriched BP terms among the upregulated genes in the cystic group were “blood vessel development”, “vasculature development” and “blood vessel morphogenesis”, “synaptic transmission”, “nervous system development” and “cell–cell signaling” in the solid group (Fig. 3a). In addition, ten pathways related with lipid metabolism were up-regulated and 9 were down-regulated in the cystic group (Fig. 3b). KEGG pathway mapping was also conducted for the DEGs to categorize their functions, and the top 10 significant pathways in the up- and downregulated genes are shown in Fig. 3c and Supplementary Table S5. Among the pathways related to lipid metabolism, “Phospholipase D signaling pathway” (28 genes; fold enrichment = 1.56; *p* < 0.05) was strongly enriched in the cystic group, whereas “Phosphatidylinositol signaling system” (12 genes; fold enrichment = 2.04; *p* < 0.01), “Regulation of lipolysis in adipocytes” (8 genes; fold enrichment = 1.89; *p* < 0.05), “Sphingolipid signaling pathway” (13 genes; fold enrichment = 1.75; *p* < 0.05), “Biosynthesis of unsaturated fatty acids” (4 genes; fold enrichment = 1.42; *p* < 0.05) and “Fatty acid elongation” (4 genes; fold enrichment = 1.30; *p* < 0.05) were enriched in the solid group (Fig. 3d). Furthermore, the transcriptomic data revealed distinct expression patterns of the genes related to lipid metabolism between the solid and cystic HBs (Fig. 3e). Finally, the expression of 6 DEGs related to lipid metabolism, including ADCY4, MGLL, ACOT2, DGKG, SHC1 and LPAR2, were confirmed by qRT-PCR (Fig. 2d). The physiological function of SCH1 is regulation of vascular endothelial functions^21^. To determine the functional significance of SHC1 in the cystic HBs, the migration ability of HUVECs was examined with SHC1 overexpression. The microscopic pictures revealed the presence of migration characteristics with SHC1-OE (Fig. 2e). The migration ability of HUVECs was also significantly enhanced by SHC1 overexpression compared to control (Fig. 2e). Taken together, cyst formation in HBs is accompanied by dysregulation of genes associated with lipid metabolism.

### Solid and cystic HBs have distinct lipidomes

**Figure 4:**
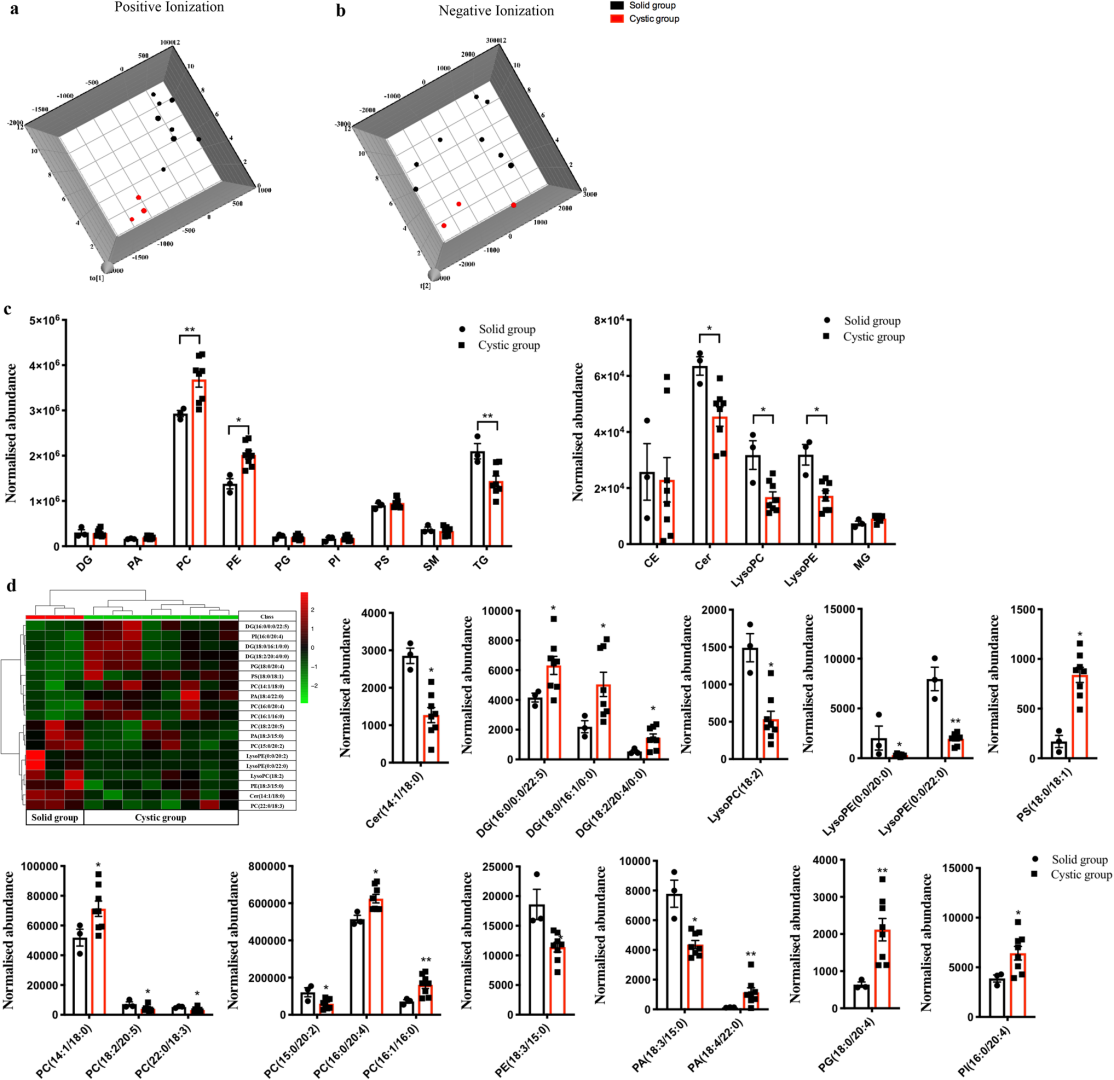
Lipidomic analysis of solid and cystic HBs. (**a**,**b**) OPLS-DA score plots. (**c**) Relative expression levels of different lipid classes in the solid and cystic HBs. *p < 0.05, **p < 0.01. (**d**) Abundance of individual lipids between solid and cystic HBs is expressed as mean ± SEM. *p < 0.05, **p < 0.01.

The lipidomic profiles of solid and cystic HBs were analyzed by UPLC-Q-TOF–MS, and the orthogonal partial least-squares discriminant analysis (OPLS-DA) plots showed significant metabolic differences between the two (Fig. 4a,b). While the relative abundance of TG, Cer, LysoPC and LysoPE was significantly reduced in the cystic HBs, that of PC and PE were increased (*p < 0.05, **p < 0.01, Fig. 4c). However, the abundance of DG, PA, PG, PI, PS, SM, CE and MG were similar in both groups (*p* > 0.05, Fig. 4c). In addition, 19 lipid compounds were significantly different between the cystic and solid HBs (Fig. 4d) on the basis of VIP > 1 and *p* < 0.05, and included Cer (14:1/18:0), DG (16:0/0:0/22:5; 18:0/16:1/0:0 and 18:2/20:4/0:0), LysoPC (18:2), LysoPE (0:0/20:0 and 0:0/22:0), PA (18:3/15:0 and 18:4/22:0), PC (14:1/18:0; 15:0/20:2; 16:0/20:4; 16:1/16:0; 18:2/20:5 and 22:0/18:3), PE (18:3/15:0), PG (18:0/20:4), PI (16:0/20:4) and PS (18:0/18:1). Although the total TG levels were significantly decreased in the cystic HBs (**p < 0.01, Fig. 4c), that of individual TGs were similar in both, and the potential target “Glycerophospholipid metabolism” pathway was strongly enriched in the cystic group (6 hits; *p* = 4.39*10^–6^).

## Discussion

HBs are benign tumors that are frequently associated with peritumoral cysts and are found throughout the CNS and can be the source of significant neurological morbidity and mortality^22^. However, the mechanisms underlying cyst formation are still poorly understood, which has hindered the development of non-surgical therapeutic strategies. HBs are an ideal tumor model to investigate the molecular mechanisms of cyst formation within the nervous system. The altered lipid metabolism and its regulatory mechanism in HB genesis and progression is of critical importance, especially in the context of cyst formation. The accompanying lipidomic changes entail a complex interplay between metabolic and transcriptional signaling pathways. Therefore, we conducted a large-scale systematic omics analysis to compare the lipidomes and transcriptomes of solid and cystic CNS HBs.

Transcriptomic analysis revealed that several genes were differentially expressed between the cystic and solid HB samples. The cystic HBs were characterized by up-regulation of genes regulating angiogenesis, which is also a key driver of tumor growth and development^23^. Since HBs are highly vascularized tumors, they are susceptible to the VEGF-induced increase in vascular permeability compared to vessels in the healthy brain^11,18^. Overexpression of VEGF has been shown in HBs and other human tumors^24^. In addition, endothelial cells respond to VEGF-A, which promotes endothelial cell proliferation and migration^25^. The Notch pathway intersects with VEGF-A signaling to coordinate endothelial cell behaviors^26^. The DEGs identified in the RNA sequence analysis included several that are directly involved in cyst formation. AQP1, an integral plasma membrane protein with six transmembrane domains, is up-regulated in cystic HBs^27^ as well as in stromal cells^28^. Additionally, previous study demonstrated epidermal growth factor receptor (EGFR) overexpression and activation in CNS HBs^29^. Expression of galectin-3 was correlated with the expression of VEGF in the development of hemangioblastoma^30^, which is a diagnostic marker and attractive molecular targets for CNS HBs. Furthermore, the DEGs were enriched in “Phospholipase D signaling pathway”, and showed a decrease in “Regulation of lipolysis in adipocytes”, “Sphingolipid signaling pathway”, “Biosynthesis of unsaturated fatty acids” and “Fatty acid elongation”, strongly suggesting that lipid metabolism plays an important role in cyst formation.

Fatty acids are critical components of cellular membrane biosynthesis. Most tumors show aberrant high rates fatty acid synthesis, elongation and desaturation that promote cancer cell proliferation^31^. Stearoyl-CoA desaturase (SCD) generates monounsaturated fatty acids in cancer cells by catalyzing the formation of double bonds at Δ9 position of palmitoyl-CoA and stearoyl-CoA^32,33^. We observed decreased levels of SCD5 in the HB tissues, which correlated with increased levels of monounsaturated fatty acids, including DG (18:0/16:1), PS (18:0/18:1) and PC (16:1/16:0). Elongates of very long chain fatty acids (ELOVLs) are involved in the elongation of fatty acids. For instance, ELOVL1 elongates the saturated C18:0–26:0 and monounsaturated C20:1n and C22:1n acyl-CoAs^34^. We observed increased ELOVL1 levels along with an increased abundance of fatty acyl chains of GLs and SLs in the cystic HBs, mainly involving DG (18:0/16:1), PS (18:0/18:1) and PA (18:4/22:0). In addition, the expression for fatty acid metabolizing enzymes like acyl-CoA thioesterases 2 (ACOT2) was down-regulated in cystic HBs, ACOT is involved in fatty acid degradation^35,36^, and its decreased levels affect the fatty acid reserves, which are alternative sources for energy production and GPs and SLs biosynthesis. A previous study showed that inhibition of fatty acid synthase (FASN) blocks HIF-1α/VEGF-A signaling in response to hypoxia, and suppresses neovascularization in glioma by upregulating VEGF165b^37^. In the present study however, FASN expression was similar in both cystic and solid HB, indicating that the higher fatty acid levels in cystic HBs are likely due to increased fatty acyls of SLs and SPs, which also promote neovascularization via VEGF signaling.

Neural cell membranes consist of glycerophospholipids that have both structural and functional roles^38^. Changes in glycerophospholipid levels affect cellular functions, endocytosis and exocytosis, cytoskeleton regulation and membrane fusion^39^. In addition, neural membrane glycerophospholipids are significantly altered in brain tumors, cognitive disorders, and neurodegenerative diseases such as Alzheimer’s disease, autism and schizophrenia^40–42^. Our results suggest that aberrant levels of lipids required for glycerophospholipid metabolism likely drive cyst formation in HBs, which was also supported by the transcriptome data.

Phosphatidylinositol 3-kinase (PI3K) is a vital regulator of multiple signaling cascades and activates the downstream targets Akt/mTOR, which is activated by the growth factors VEGF^43^. Consistent with this, the genes involved in the PI3K signaling pathway were also dysregulated in our study. The cystic HBs showed increased expression of inositol polyphosphate-4-phosphatase type II B and phosphoinositide-3-kinase regulatory subunit 5, and decreased levels of inositol 1,4,5-trisphosphate receptor type 1, inositol polyphosphate-4-phosphatase type I A, myotubularin related protein 1, inositol polyphosphate-5-phosphatase J, phosphatidylinositol-4,5-bisphosphate 3-kinase catalytic subunit beta and diphosphoinositol pentakisphosphate kinase 1. In contrast, the total PI content was similar in both HB types, while the significantly different PI species were mostly at low abundance in the cystic HBs as per the lipidomics results. The occurrence of cystic HBs is frequently associated with molecular pathway changes in epidermal growth factor receptor and PI3K/Akt/mTOR pathways^44^. However, the exact role of PI3K signaling in cystic HBs formation still needs to be elucidated.

In conclusion, cyst formation in CNS tumors is associated with aberrant lipid biosynthesis, glycerophospholipid metabolism and phospholipase activity. Our results give novel insights into the cyst formation in HBs in the context of aberrant lipid metabolism.

## Materials and methods

### Human tissue samples collection

Surgically resected brain tissue samples were collected from 11 HB patients—8 cystic and 3 solid tumor cases—from the West China Hospital of Sichuan University. The following clinical information was also collected for each patient: age, gender, duration of illness, symptoms, tumor characteristics, location, size and VHL disease (Table 1).

### Statements

The study was approved by the ethics committee of the West China Hospital of Sichuan University, and all experiments were performed in accordance with relevant guidelines and regulations. Informed consent was obtained from all subjects or, if subjects are under 18 years of age, from a parent and/or legal guardian.

### Cell culture and generation of SHC1-overexpression HUVECs (SHC-OE)

Human umbilical vein endothelial cells (HUVEC) from the American Type Culture Collection (ATCC, Manassas, VA, USA) were cultured in Dulbecco's modified Eagle's medium (DMEM) supplemented with 10% fetal bovine serum (FBS; Hyclone; UT, USA).

HUVEC expressing increased levels of the SCH1 protein were generated as described previously^45^. Briefly, the gene of SHC1 (NM_183001.5) was cloned into the shuttle vector pSLenti-EF1-EGFP-P2A-Puro-CMV-MCS-3xFLAG-WPRE. The reconstructed plasmid was verified by Sanger sequencing. For lentivirus packing, HEK293T cells (ATCC, Manassas, VA, USA) were co-transfected with a control vector or vector carrying SCH1 fragment, and lentiviral packaging mix using Lipofectamine 2000 (Invitrogen, Carlsbad, CA, USA). Supernatants were collected 48 h after transfection and centrifuged at 300×*g* for 10 min to remove cell debris and filtered through a 0.45 μm pore-size filter. The lentiviral particles were concentrated by ultracentrifugation for 90 min at 50,000×*g* and 4 °C. Concentrated lentivirus was stored at − 80 °C. Genetic modified HUVECs were used in the following assays 72 h after lentiviral infection. SHC1 mRNA expression level in control and SHC1-overexpressed HUVECs (SHC1-OE) were measured by RT-qPCR.

### In vitro scratch wound assay

HUVECs and SHC1-OE cells were plated in equal numbers in 6-well tissue culture plates (3 × 10^5^ cells/well) to achieve 90% confluence. Thereafter, a vertical wound was created using a 200 μL pipette tip within a certain area. Scraped cells were removed by washing the monolayer twice with PBS. The cells were incubated in DMEM containing with 1% FBS at 37 °C in 5% CO_2_ for 72 h. The area of the cell-free wound was recorded with microscopy at 0, 24, 48 and 72 h. Measurement of scratch wound was assessed by using ImageJ software.

### Immunohistochemical staining

Hematoxylin–eosin (HE) and immunohistochemical staining were performed by standard techniques as previously described. The following primary antibodies were used: anti-CD34 (ab81289; Abcam), anti-NSE (PA5-27,452; Invitrogen), anti-D2-40 (MA1-83,884; Invitrogen), anti-CA9 (ab184006; Abcam), anti-PCK (ab28455; Abcam), anti-Oligo2 (ab109186; Abcam), anti-SOX9 (#82,630; Cell signaling technology), anti-Inhibin (PA5-81,202; Invitrogen), anti-GFAP (ab7260; Abcam), anti-S100 (ab183979; Abcam), and anti-Ki67 (ab15580; Abcam).

### RNA isolation and High-throughput sequencing

Total RNA was isolated from 3 samples each of solid and cystic tumors using the TRIzol reagent (Invitrogen, Carlsbad, CA, USA). The RNA concentration was measured using a NanoDrop ND-2000 instrument (Thermofisher Scientific), and its integrity was evaluated by denaturing agarose gel electrophoresis.

Briefly, the rRNAs were removed from the total RNA mix using Ribo-Zero rRNA Removal Kit (Illumina, San Diego, CA, USA), and the RNA-seq libraries were constructed using the TruSeq Stranded Total RNA Library Prep Kit (Illumina, San Diego, CA, USA). Quality control of the libraries was performed using Agilent 2100 Bioanalyzer (Agilent, Santa Clara, CA) and sequenced using an Illumina HiSeq Sequencer with a 150 bp paired-end run.

### Bioinformatics analysis

The Gene Ontology (GO) and Kyoto Encyclopedia of Genes and Genomes (KEGG) pathway enrichment analyses (https://www.genome.jp/kegg, accessed on December 13th 2018), were performed for the differentially expressed genes (DEGs) to interpret their biological functions^46,47^. The top 10 enriched GO terms and the significant KEGG pathways were identified on the basis of −log10 *p*-value.

### Quantitative reverse transcription-PCR (qRT-PCR) analysis

Randomly selected genes were validated by qRT-PCR. Total RNA was reverse transcribed into cDNA using a kit (Invitrogen, Carlsbad, CA, USA), and q-PCR was performed on a Bio-rad CFX Connect Real-Time PCR System. The relative expression levels of target genes were normalized to *gapdh* using the 2^−∆∆Ct^ method. All primers are listed in Supplementary Table S1.

### Accession numbers

RNA-seq data are available at the Gene Expression Omnibus (GEO) database repository under the accession number GSE148216.

### Lipid extraction and LC–MS lipid metabolite analysis

Total lipids were extracted from ~ 10 mg frozen tissue using methyl-tert-butyl ether (MTBE) as previously described^48^. Briefly, 150 µl ice-cold methanol was added to each brain tissue sample (25–30 mg) followed by 450 µl MTBE, and then mixed gently. The samples were incubated at 4 °C for 10 min on an orbital shaker, and homogenized by ultra-sonication for another 10 min on an ice bath. To separate the lipid and aqueous phases, 300 μl of 25% methanol was added to the homogenates and were mixed vigorously. After centrifuging at 14,000*g* at 4 °C for 10 min, 500 μl of the upper lipid layer was aspirated, dried under a gentle stream of nitrogen and stored at − 80 °C until use.

Chromatography was performed on the UPLC-ESI-Q-TOF–MS (Waters) platform using ACQUITY UPLC HSS T3 column (1.8 μm, 2.1 × 100 mm; Waters). The MS was operated in both the positive (+ ESI) and negative (-ESI) modes using G2-S Q-tof mass spectrometer since some lipid species are only detected in one ionization mode. The data were collected in the continuous mode using MassLynx (Version 4.1, Waters).

### Data processing and analysis

UPLC-ESI-Q-TOF–MS acquisition data was submitted and the alignment, peak selection and identification of lipids were performed by the Progenesis QI software (Version 2.0, Waters) according to the manufacturer’s instructions. The metabolites were identified against the Lipid Maps Database (www.lipidmaps.org) and the Human Metabolome Database (http://www.hmdb.ca/), and the lipid molecules with the highest impact on the group clustering were identified in the variable importance (VIP)-plots (VIP > 1). The significance of each metabolite was determined on the basis of the chemical shift (unpaired Student’s t-test p < 0.05). The metabolites with VIP > 1 and *p* < 0.05 were identified as significantly different between the groups.

### Statistical analysis

All data are represented as the mean ± standard error (SEM). The two groups were compared by unpaired two-tailed Student's t-test, and *p* values < 0.05 were considered statistically significant. All statistical analyses were performed with GraphPad Prism version 8.4.0 (GraphPad, San Diego, CA).

## Supplementary Information

Supplementary Information 1.

Supplementary Information 2.

Supplementary Information 3.

Supplementary Information 4.

Supplementary Information 5.

## Abbreviations

DG: Diacylglycerol
PC: Phosphatidylcholine
PE: Phosphatidylethanolamine
PI: Phosphatidylinositol
PS: Phosphatidylserine
SM: Sphingomyelin
TG: Triacylglycerol
PGP: Phosphatidylglycerolphosphate
MG: Monoradylglycerol
CerP: Ceramide 1-phosphate
GlcCer: Glucosylceramide
CE: Cholesterylester
Cer: Ceramide
LacCer: Lactosylceramide
LysoPC: Lysophosphatidylcholine
LysoPE: Lysophosphatidylethanolamine
MGDG: Monogalactosyldiacylglycerol
PA: Phosphatidic acid
PG: Phosphatidylglycerol
