# Supplementary material for: Combined transcriptomic and lipidomic analysis reveals aberrant lipid metabolism in central nervous system hemangioblastomas
Source: Sci Rep. 2021 Jan 14;11:1314. doi: 10.1038/s41598-020-80263-8 (PMC7809491; doi:10.1038/s41598-020-80263-8)
Supplement: Supplementary file 1 — Supplementary Information 1. [file 41598_2020_80263_MOESM1_ESM.docx]

Table S1. List of RT-PCR primers were used to validate the RNA sequence results.

| **Gene** | **Primer (5'to3')** |
| --- | --- |
| ADCY4-F | GCCTCGCCTCCTCACTC |
| ADCY4-R | ACTCCTGCCACGTTCCC |
| ACOT2-F | CATCTCCTGCTGTCCTTCG |
| ACOT2-R | ATCTGACCAACCTGCCTCA |
| DGKG-F | TGACCCTCATAGCAACCTC |
| DGKG-R | TCCCCTGACTAACCATGTG |
| LPAR2-F | CTACCTGCTCGGCAATCTG |
| LPAR2-R | AAGTGAAAGTCGGGCTGTG |
| MGLL-F | CACCAACTCCGTCTTCCA |
| MGLL-R | CCATCTCTGCCCTTCCC |
| SHC1-F | CCTCCACCTCCCCAGTC |
| SHC1-R | GGTCGTGCTCTCCCGTA |
| GAPDH-F | AACATCATCCCTGCCTCTACTG |
| GAPDH-R | TGCTTCACCACCTTCTTGATGT |
