# Supplementary material for: Combined transcriptomic and lipidomic analysis reveals aberrant lipid metabolism in central nervous system hemangioblastomas
Source: Sci Rep. 2021 Jan 14;11:1314. doi: 10.1038/s41598-020-80263-8 (PMC7809491; doi:10.1038/s41598-020-80263-8)
Supplement: Supplementary file 2 — Supplementary Information 2. [file 41598_2020_80263_MOESM2_ESM.docx]

**Table 2. Immunohistochemical expression in HBs patients.**

| Case No | NSE | D2-40 | Inhibin | CD34 | CA9 | PCK | Oligo2 | SOX9 | GFAP | S100 | Ki67 |
| --- | --- | --- | --- | --- | --- | --- | --- | --- | --- | --- | --- |
| 1 | + | + | - | + | NA | NA | NA | NA | - | ± | 1-5% |
| 2 | + | + | - | NA | NA | - | - | NA | - | NA | 30% |
| 3 | + | + | - | + | NA | NA | NA | NA | - | + | NA |
| 4 | + | + | - | NA | + | - | NA | NA | NA | + | 5% |
| 5 | + | NA | + | + | NA | - | - | NA | - | - | 10% |
| 6 | + | - | - | + | + | NA | NA | NA | - | NA | 2% |
| 7 | + | ± | ± | NA | + | - | NA | NA | - | NA | NA |
| 8 | + | - | - | NA | NA | NA | - | + | - | + | 2%-3% |
| 9 | + | + | + | NA | NA | NA | - | + | - | NA | 2% |
| 10 | + | - | - | + | NA | NA | NA | NA | - | NA | 2% |
| 11 | + | + | - | + | NA | NA | NA | NA | - | NA | NA |

+: High degree of positive; ±: Low degree of positive; -: Negative; NA: Not application
